# Supplementary material for: New Insights on Lilium Phylogeny Based on a Comparative Phylogenomic Study Using Complete Plastome Sequences
Source: Plants (Basel). 2019 Nov 27;8(12):547. doi: 10.3390/plants8120547 (PMC6963401; doi:10.3390/plants8120547)
Supplement: Supplementary file 1 [file plants-08-00547-s001.pdf]

Supplementary Material

Table S1. Summary of three plastome sequences.

Taxon	Length (bp)				GC Content (%)			
	LSC	SSC	IR	Total	LSC	SSC	IR	Total
Lilium candidum	81,481	17,644	26,488	152,101	34.9	30.6	42.5	37.0
Lilium formosanum	82,101	17,524	26,514	152,653	34.9	30.8	42.4	37.0
Lilium leichtlinii var. *maximowiczii*	82,037	17,583	26,492	152,604	34.8	30.7	42.5	37.0

Table S2. List of estimated species trees according to different options.

Data Set	Type	Tool	Method	Modeltest	Tree Names
All_loci (189)	Partitioned	RAxML	Maximum-likelihood analysis	PartitionFinder 2	ALL_part_RAxML
		IQ-TREE	Maximum-likelihood analysis	ModelFinder	ALL_part_iqtree
		MrBayes	Bayesian inference	PartitionFinder 2	ALL_part_mrbayes
	Non-partitioned	RAxML	Maximum-likelihood analysis	Jmodeltest 2	ALL_nonpart_RAxML
		IQ-TREE	Maximum-likelihood analysis	Jmodeltest 2	ALL_nonpart_iqtree
		MrBayes	Bayesian inference	Jmodeltest 2	ALL_nonpart_mrbayes
CDSs (78)	Partitioned	RAxML	Maximum-likelihood analysis	PartitionFinder 2	CDSs_part_RAxML
		IQ-TREE	Maximum-likelihood analysis	ModelFinder	CDSs_part_iqtree
		MrBayes	Bayesian inference	PartitionFinder 2	CDSs_part_mrbayes
	Non-partitioned	RAxML	Maximum-likelihood analysis	Jmodeltest 2	CDSs_nonpart_RAxML
		IQ-TREE	Maximum-likelihood analysis	Jmodeltest 2	CDSs_nonpart_iqtree
		MrBayes	Bayesian inference	Jmodeltest 2	CDSs_nonpart_mrbayes
IGSs (90)	Partitioned	RAxML	Maximum-likelihood analysis	PartitionFinder 2	IGSs_part_RAxML
		IQ-TREE	Maximum-likelihood analysis	ModelFinder	IGSs_part_iqtree
		MrBayes	Bayesian inference	PartitionFinder 2	IGSs_part_mrbayes
	Non-partitioned	RAxML	Maximum-likelihood analysis	Jmodeltest 2	IGSs_nonpart_RAxML
		IQ-TREE	Maximum-likelihood analysis	Jmodeltest 2	IGSs_nonpart_iqtree
		MrBayes	Bayesian inference	Jmodeltest 2	IGSs_nonpart_mrbayes
Introns	Partitioned	RAxML	Maximum-likelihood analysis	PartitionFinder 2	INTRONs_part_RAxML

(21)		IQ-TREE	Maximum-likelihood analysis	ModelFinder	INTRONs_part_iqtree
		MrBayes	Bayesian inference	PartitionFinder 2	INTRONs_part_mrbayes
	Non-partitioned	RAxML	Maximum-likelihood analysis	Jmodeltest 2	INTRONs_nonpart_RAxML
		IQ-TREE	Maximum-likelihood analysis	Jmodeltest 2	INTRONs_nonpart_iqtree
		MrBayes	Bayesian inference	Jmodeltest 2	INTRONs_nonpart_mrbayes

Estimated Species Trees Using Gene Trees by ASTRAL

**Data**	**Tree Names**
189 gene trees from CDSs, IGSs, and introns	ASTRAL_ALL
78 gene trees from CDSs	ASTRAL_CDSs
90 gene trees from IGSs	ASTRAL_IGSs
21 gene trees from introns	ASTRAL_INTRONs

Table S3. 189 loci used for phylogenetic analyses in this paper.

Data Set	Locus	Length	Data Set	Locus	Length	Data Set	Locus	Length
	accD	1584		*psaJ*	141		*rps4*	612
	atpA	1524		*psbA*	1062		*rps7*	468
	atpB	1503		*psbB*	1527		*rps8*	429
	atpE	408		*psbC*	1422		*rps11*	423
	atpF	570		*psbD*	1062		*rps12*	372
	atpH	246		*psbE*	252		*rps14*	303
	atpI	744		*psbF*	120	CDS	*rps15*	273
	ccsA	972		*psbH*	222		*rps16*	252
	cemA	702		*psbI*	111		*rps18*	306
	clpP	621		*psbJ*	123		*rps19*	279
	matK	1557		*psbK*	192		*ycf1*	5631
	ndhA	1095		*psbL*	117		*ycf2*	6798
	ndhB	1533		*psbM*	105		*ycf3*	513
	ndhC	363		*psbN*	132		*ycf4*	555
	ndhD	1510		*psbT*	102		*accD-psaI*	1061
CDS[a]	*ndhE*	308	CDS	*psbZ*	189		*atpB-rbcL*	876
	ndhF	2308		*rbcL*	1443		*atpF-atpH*	582
	ndhG	547		*rpl2*	825		*atpH-atpI*	1098
	ndhH	1182		*rpl14*	369		*atpI-rps2*	301
	ndhI	543		*rpl16*	408		*ccsA-ndhD*	194
	ndhJ	477		*rpl20*	361		*cemA-petA*	261
	ndhK	791		*rpl22*	412		*clpP-psbB*	522
	petA	963		*rpl23*	282	IGS[b]	*ndhB-rps7*	314
	petB	654		*rpl32*	195		*ndhC-trnV*	1121
	petD	504		*rpl33*	204		*ndhD-psaC*	143
	petG	114		*rpl36*	114		*ndhE-ndhG*	235
	petL	96		*rpoA*	1088		*ndhF-rpl32*	791
	petN	90		*rpoB*	3207		*ndhG-ndhI*	409
	psaA	2253		*rpoC1*	2079		*ndhH-rps15*	137
	psaB	2205		*rpoC2*	4227		*ndhJ-ndhK*	187
	psaC	246		*rps2*	711		*petA-psbJ*	921

psaI	105	*rps3*	657	*petB-petD*	216

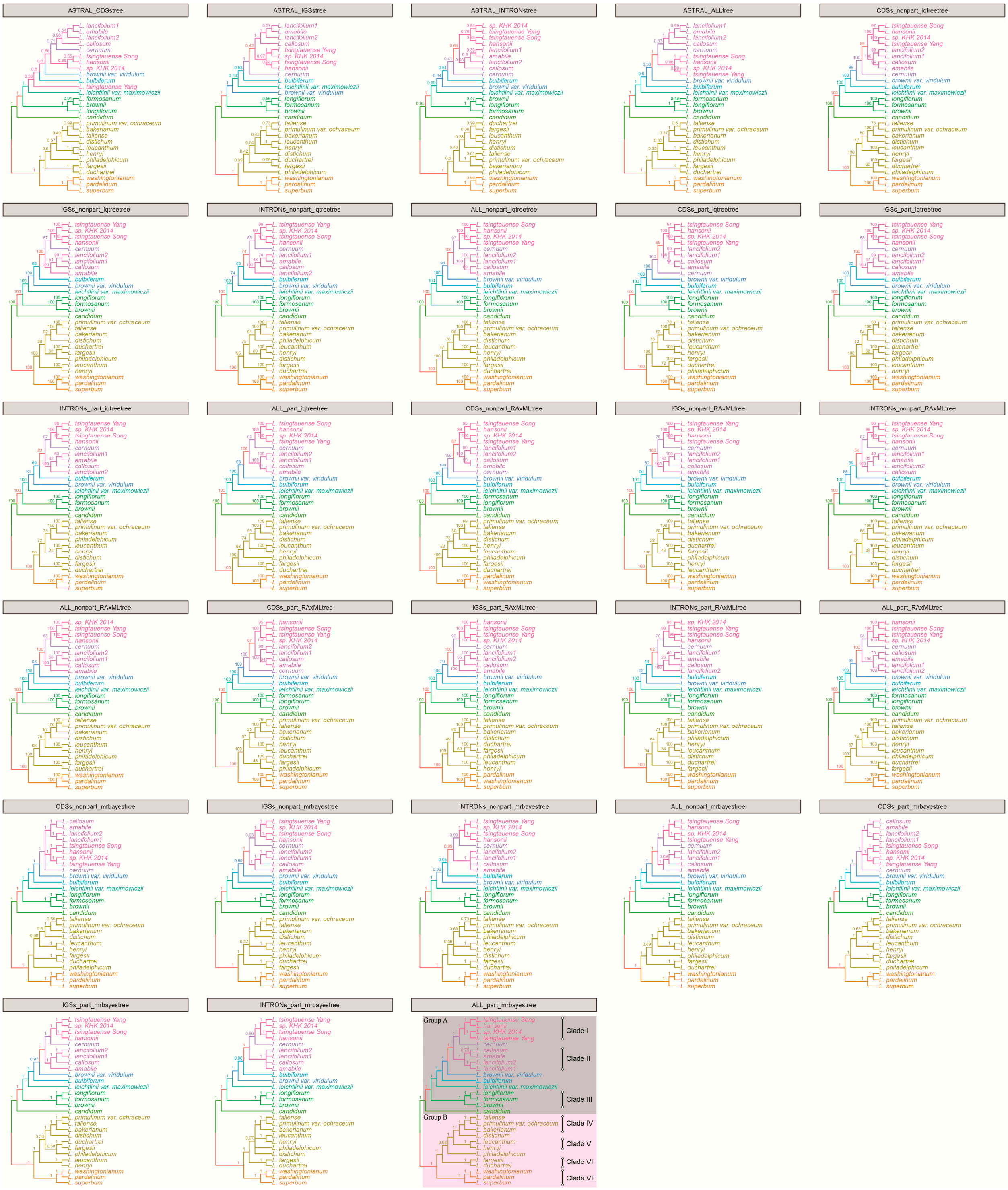

Figure S1. A clade of *Lilium* species extracted from 28 species trees.

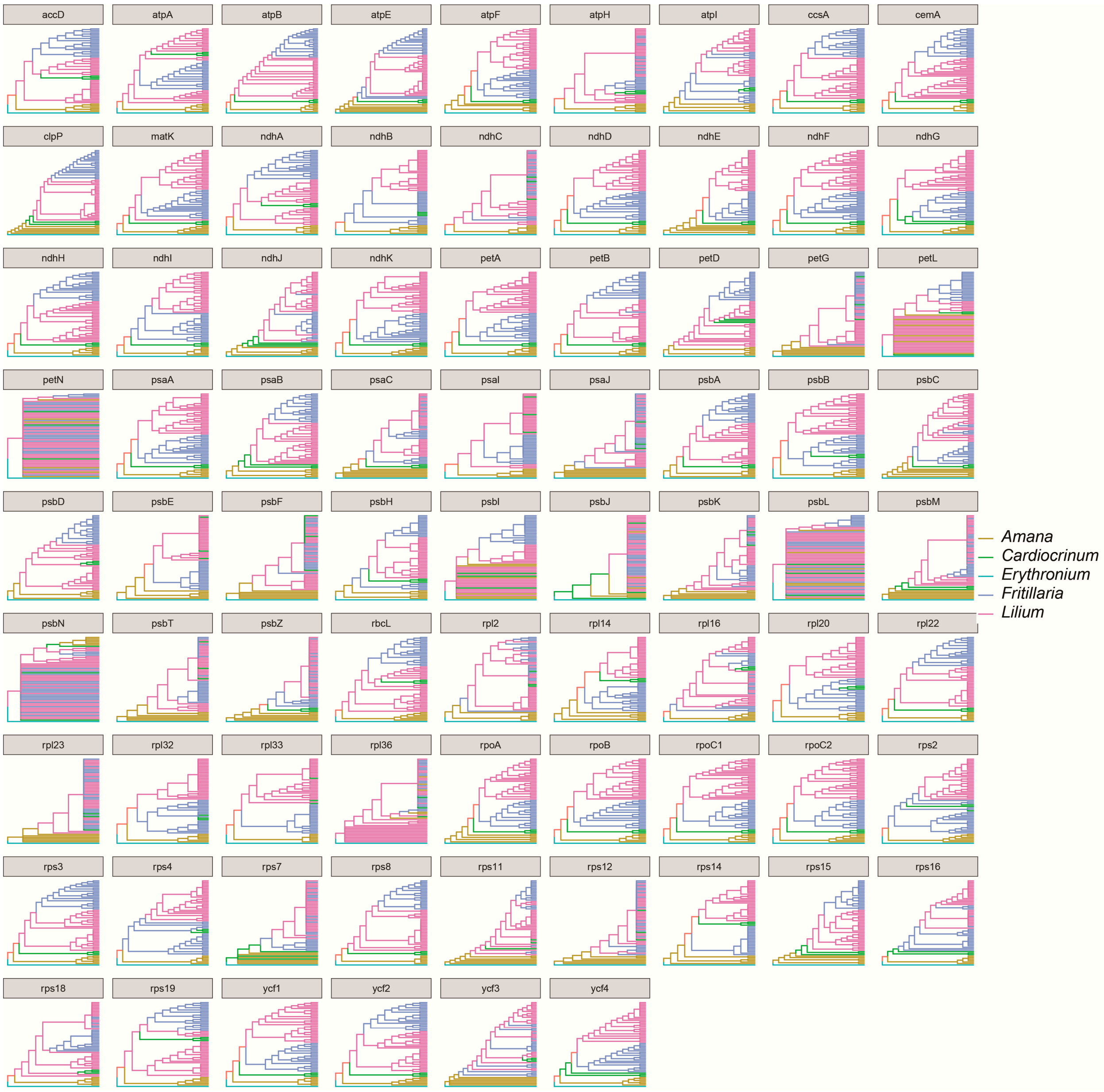

Figure S2. Gene trees constructed using 78 coding regions. Coloured line refers to each genus.

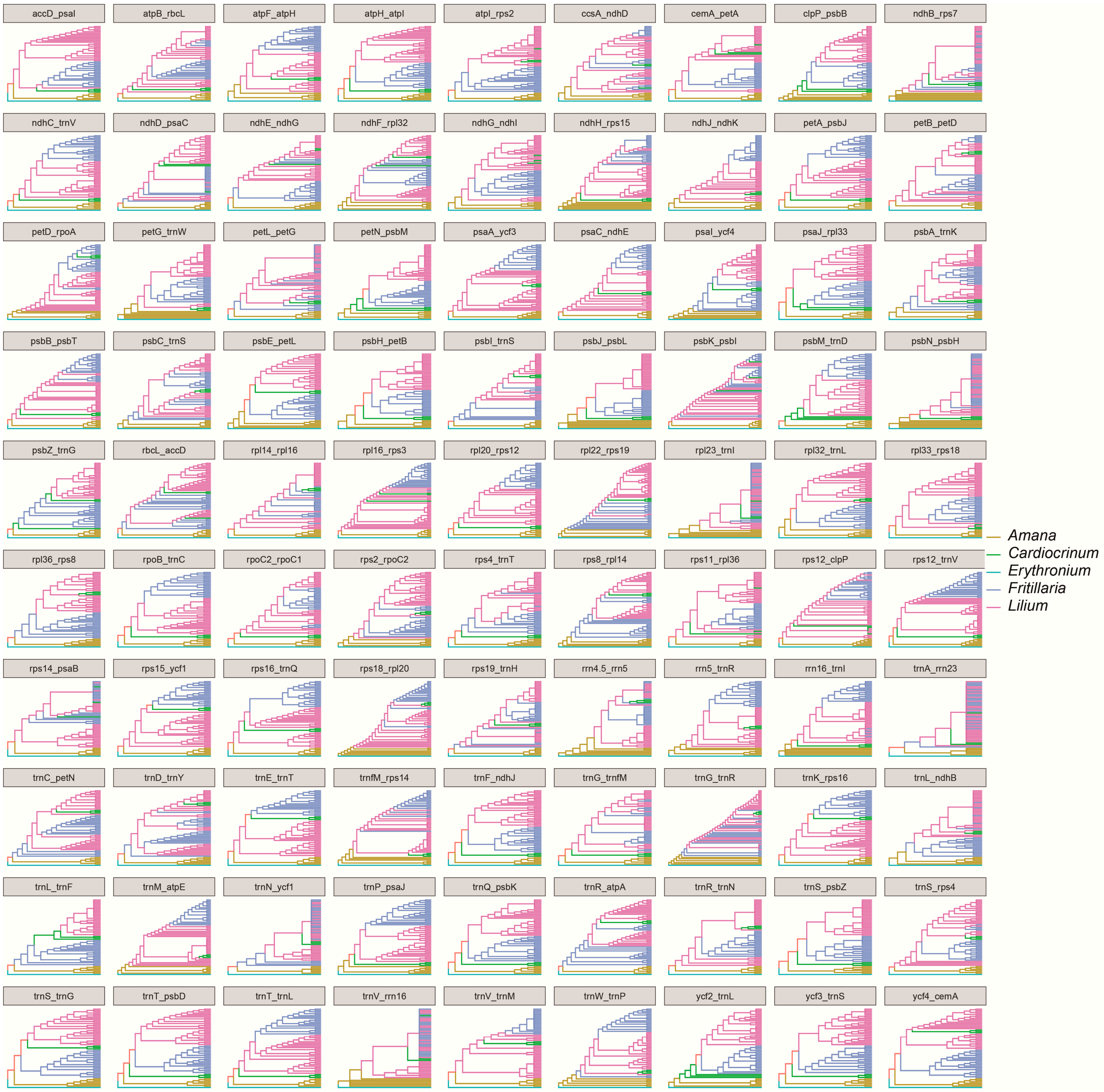

Figure S3. Gene trees constructed using 90 intergenic spacers. Coloured line refers to each genus.

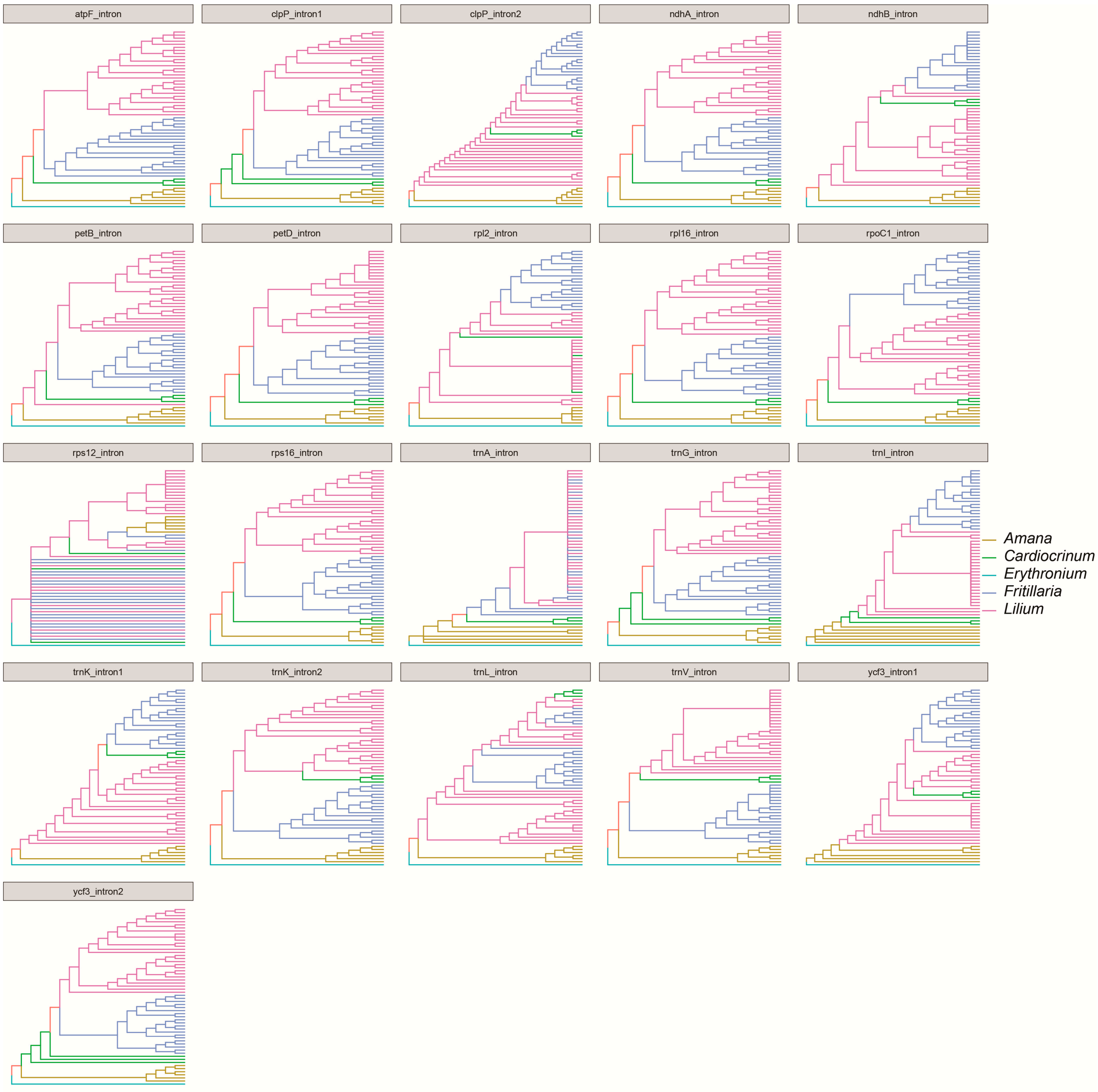

Figure S4. Gene trees were constructed using 21 introns. Coloured line refers to each genus.

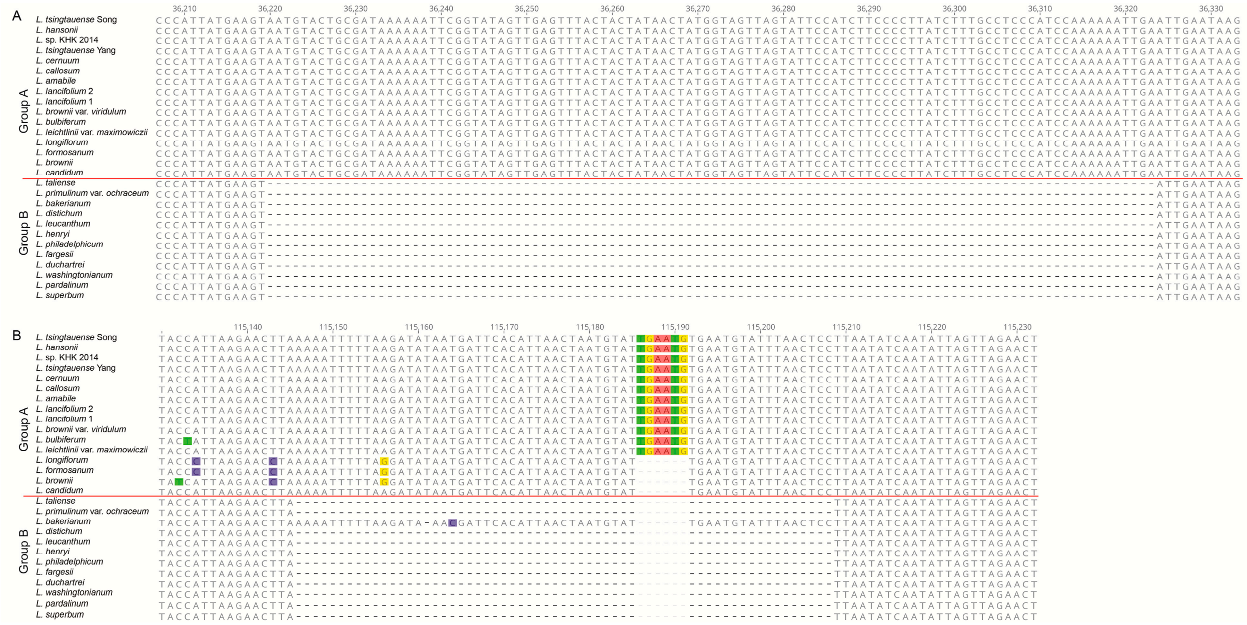

Figure S5. Large indertions/deletions occurring at least five species with longer than 50 bp. Red line distinguishes between two groups.
